# CanSino COVID-19 Vaccine: Comparison of Vaccine Adverse Effects Among Diabetic and Non-diabetic Recipients

**DOI:** 10.7759/cureus.47391

**Published:** 2023-10-20

**Authors:** Shahad Abduljalil Abualhamael, Atif A Hashmi

**Affiliations:** 1 Internal Medicine, Faculty of Medicine in Rabigh, King Abdulaziz University, Jeddah, SAU; 2 Pathology, Liaquat National Hospital and Medical College, Karachi, PAK

**Keywords:** covid vaccination in pakistan, covid-19 vaccine complication, covid-19, swelling, pain, injection site, fever, vaccine, cansino

## Abstract

Introduction

The emergence of potent vaccines is crucial in the fight against the coronavirus disease 2019 (COVID-19) pandemic. Two of the many factors influencing the acceptance of the vaccine are perceptions about its efficacy, effectiveness, safety, and side effects. Thus, this study compared patients with and without diabetes mellitus (DM) who received the CanSino (CanSinoBIO, Tianjin, China) COVID-19 vaccination to identify the prevalence of local and general side effects.

Methods

This was a multicenter, cross-sectional study performed using a non-probability sampling method. The study period was six months, from August 1, 2022, to January 31, 2023. The study included 600 participants who provided informed consent and had received the CanSino vaccine in a single dose. Demographic characteristics of the participants, including gender, age, weight, and height; comorbidities such as hypertension and diabetes; previous infection with COVID-19; and the prevalence of any local and systemic side effects following vaccination, were documented. Between diabetic and non-diabetic participants, the relationship between local and general side effects and satisfaction levels was assessed using the chi-square test.

Results

The study findings showed that out of 600 participants, 287 (95.7%) were males and 13 (4.3%) were females who had DM, whereas 229 (76.3%) males and 71 (23.7%) females did not. There was a statistically significant association between the two groups (p < 0.001). After receiving a single dose of the CanSino vaccine, the most frequently noticeable side effect was fever, which was noticed in 260 (86.75%) diabetic patients and 279 (93.0%) non-diabetic participants, with a significant association noted among them (p=0.010). Among the non-diabetic participants, 164 (54.7%) were satisfied, and 155 (51.7%) diabetics and 65 (21.7%) non-diabetic participants were extremely pleased with their vaccinations.

Conclusion

This study concluded that participants with comorbid diseases such as DM had both general and local side effects far more frequently than those without DM. The most noticeable side effects after a single dose of CanSino were fever, injection site pain, and burning. The CanSino vaccine did not require hospitalization and had a relatively low frequency of local and systemic side effects.

## Introduction

The identification of coronavirus disease 2019 (COVID-19) and its dissemination has been extremely worrisome for people around the world. Vaccination is one of the proposed ways to combat the pandemic and reduce the severity of infection rates [1]. In February 2021, Pakistan began its immunization campaign using the Sinopharm vaccine from China. Consequently, many vaccines, including Sinovac, CanSino, Sputnik, AstraZeneca, Pfizer-BioNTech, and Moderna, have been approved and are now being widely used throughout the nation. [2]. CanSino (CanSinoBIO, Tianjin, China) is a recombinant viral vector vaccine developed by China. It uses adenovirus as a vector to transfer the genes that code for COVID-19 proteins to human cells, resulting in the production of deactivating antibodies against severe acute respiratory syndrome coronavirus 2 (SARS-CoV-2). It requires only one intramuscular dose and is often effective [3]. Limited evidence suggests that the CanSino vaccine is comparable with other viral vector vaccines but less immunogenic than messenger ribonucleic acid (mRNA) vaccines [4]. In addition, phase III clinical trial findings at 28 days following CanSino immunization showed a vaccine efficacy of 58% [5], whereas a retrospective cohort analysis from China produced a vaccine effectiveness estimate of 61.5% [6].

Diabetes mellitus (DM) is a significant concern in healthcare worldwide because of its high mortality and morbidity. With a more serious illness, less favorable results, and an elevated death rate, underlying DM is a substantial risk factor for being more susceptible to COVID-19 [7]. Recent studies have shown that people with type 2 and type 1 DM are more likely than those without DM to experience severe COVID-19 illness. [8].

Patients with DM are more likely to have severe symptoms after a virus-related infection and are approximately three times more likely to die of COVID-19. Patients with DM are given attention for early vaccinations because they often experience worse outcomes than people without diabetes [9]. Numerous vaccinations have been approved to protect against the COVID-19 infection. The vaccines provided by Johnson & Johnson, Moderna, Pfizer, and BioNTech are reportedly effective and safe for diabetic patients [10]. The Oxford/AstraZeneca vaccine has been linked to rare but serious side effects, namely the formation of blood clots [9-11]. 

Data on the incidence of chronic diseases such as DM in nations with limited resources, such as Pakistan, are currently scarce. Before administering COVID-19 vaccinations, doctors must be aware of any potential adverse effects or problems in patients with DM. Therefore, this study compared patients with and without DM who received the CanSino COVID-19 vaccination to detect the incidence of local and general side effects.

## Materials and methods

This multicenter, cross-sectional study was conducted using a non-probability sampling technique. The study duration was six months, from August 1, 2022, to January 31, 2023. Ethical approval for the study was obtained from Essa General Hospital, Karachi, Pakistan (approval number: Essa/37/2022). There were 600 participants in the study who provided informed consent and received the CanSino vaccination in a single dose. Participants who had received a vaccination with a different vaccine (other than CanSino) or who had never received even one dose of the COVID-19 vaccine were excluded from the study. In addition, participants with an active COVID-19 infection were excluded, along with patients with immunodeficiency and severe underlying illnesses.

The participants’ basic demographic information was collected using a structured proforma at the time of vaccination. The demographic characteristics of the participants, including gender, age, weight, and height; comorbidities such as hypertension and DM; and previous infection with COVID-19, were recorded. The duration of DM was also documented in diabetic patients. The prevalence of local side effects, such as pain, soreness, swelling, rashes, and burning at the injection site, and general side effects post-vaccination, were documented twice: once at one week and then six weeks following the vaccination. The participants’ level of satisfaction was also recorded.

Data were entered and analyzed using IBM SPSS Statistics software for Windows, Version 26.0 (IBM Corp., Armonk, NY). Age, height, weight, hypertension, and diabetes duration were all given as means and standard deviations. Demographic characteristics, including gender, frequency, and proportion of both local and general side effects, were recorded. The association between the mean and standard deviations of age, weight, height, and duration of DM and hypertension was determined using an independent t-test. Between diabetic and non-diabetic participants, the relationship between local and general side effects and satisfaction levels was assessed using the chi-square test. A p-value of <0.05 was considered statistically significant.

## Results

A total of 600 participants who received a single dose of the CanSino vaccination were included in the study; of them, 287 (95.7%) were males and 13 (4.3%) were females who had DM, whereas 229 (76.3%) males and 71 (23.7%) females were non-diabetic, with a statistically significant association between these two groups (p<0.001). The mean age of the diabetic individuals was 36.94 ±11.97 years, whereas that of the non-diabetic participants was 40.50±16.91 years, with a statistically significant association between them (p=0.003). The mean weight of the diabetic participants was 66.26±13.35 kg, whereas the non-diabetic participants had a mean weight of 71.34±20.66 kg, displaying a significant association (p<0.001). Individuals with DM had a mean height of 5.47±0.61 feet, whereas those without diabetes had a mean height of 5.39±0.75 feet. This difference in mean heights was not significant (p=0.137). The mean duration of hypertension was 2.80±0.40 years in diabetics and 2.50±0.50 years in non-diabetics, with a significant difference (p=0.001). The mean duration of DM was 1.82±0.68 years. Of the 600 patients who were diabetics and non-diabetics, 139 (46.3%) and 136 (45.3%) had hypertension, respectively, with a statistically insignificant difference (p=0.806). Furthermore, it was found that neither subjects with DM nor those without DM had ever had a COVID-19 infection, as presented in Table 1.

**Table 1:** Demographic particulars of diabetic and non-diabetic participants (n=600) SD: standard deviation Data have been represented as *Mean±SD; **p-value significant as <0.05; ***data have been represented as n(%)

Variables		Mean±SD/n(%)		p-value
		Diabetes mellitus		
		Yes	No	
Age (years)*		36.94±11.97	40.50±16.91	0.003**
Weight (kg)*		66.26±13.35	71.34±20.66	<0.001**
Height (feet)*		5.47±0.61	5.39±0.75	0.137
Hypertension, duration (years)*		2.80±0.40	2.50±0.50	0.001**
Gender***	Male	287 (95.7%)	229 (76.3%)	<0.001**
	Female	13 (4.3%)	71 (23.7%)	
Hypertension***	Yes	139 (46.3%)	136 (45.3%)	0.806
	No	161 (53.7%)	164 (54.7%)	
Previous COVID-19 infection***	Yes	0 (0.0%)	0 (0.0%)	NA
	No	300(100.0%)	300 (100.0%)	

After receiving a single dose of the CanSino vaccine, the most commonly noticeable side effect was fever, which was noticed in 260 (86.75%) diabetic patients and 279 (93%) non-diabetic participants, with a significant relationship noted between them (p=0.010). Moreover, 146 (48.7%) non-diabetic participants and 255 (85%) diabetic patients had injection site burning, with a significant difference (p<0.001). Likewise, a significant association (p<0.05) was observed between the diabetic and non-diabetic participants in terms of several adverse effects, including injection site pain, redness, lymphadenopathy, nausea, anxiety, tiredness, muscular pain, cough, and chest pain. Conversely, in both diabetic and non-diabetic participants, an insignificant association was found in terms of injection site swelling, headaches, rashes, flu-like illness, joint pain, chills, sore throat, and diarrhea (p>0.05). Shortness of breath was reported at low frequency in the diabetic group, whereas 102 (34%) cases in the non-diabetic group reported shortness of breath, with a significant difference (p<0.001), as presented in Table 2.

**Table 2:** Association of CanSino vaccine adverse effects with diabetes mellitus *Data have been represented as n(%); **p-value significant as <0.05

Variable*		n(%)		p-value
		Diabetes mellitus		
		Yes	No	
Pain at the site of injection	Yes	200 (66.7%)	123 (41.0%)	<0.001**
	No	100 (33.3%)	177 (59.0%)	
Swelling at the site of injection	Yes	150 (50.0%)	141 (47.0%)	0.462
	No	150 (50.0%)	159 (53.0%)	
Redness at the site of injection	Yes	110 (36.7%)	164 (54.7%)	<0.001**
	No	190 (63.3%)	136 (45.3%)	
Lymphadenopathy	Yes	198 (66.0%)	27 (9.0%)	<0.001**
	No	102 (34.0%)	273 (91.0%)	
Fever (temperature >37.8 ˚C)	Yes	260 (86.75)	279 (93.0%)	0.010**
	No	40 (13.3%)	21 (7.0%)	
Headache	Yes	132 (44.0%)	133 (44.3%)	0.934
	No	168 (56.0%)	167 (55.7%)	
Nausea	Yes	57 (19.0%)	28 (9.3%)	0.001**
	No	243 (81.0%)	272 (90.7%)	
Rashes	Yes	153 (51.0%)	163 (54.3%)	0.414
	No	147 (49.0%)	137 (45.7%)	
Burning at the injection site	Yes	255 (85.0%)	146 (48.7%)	<0.001**
	No	45 (15.0%)	154 (51.3%)	
Flu	Yes	45 (15.0%)	45 (15.0%)	1.000
	No	255 (85.0%)	255 (85.0%)	
Anxiety	Yes	255 (85.0%)	102 (34.0%)	<0.001**
	No	45 (15.0%)	198 (66.0%)	
Myalgia	Yes	85 (28.3%)	47 (15.7%)	<0.001**
	No	215 (71.7%)	253 (84.3%)	
Fatigue	Yes	110 (36.7%)	161 (53.7%)	<0.001**
	No	190 (63.3%)	139 (46.3%)	
Joint pain	Yes	143 (47.7%)	162 (54.0%)	0.121
	No	157 (52.3%)	138 (46.0%)	
Chills	Yes	149 (49.7%)	163 (54.3%)	0.253
	No	151 (50.3%)	137 (45.7%)	
Cough	Yes	138 (46.0%)	47 (15.7%)	<0.001**
	No	162 (54.0%)	253 (84.3%)	
Sore throat	Yes	87 (29.0%)	88 (29.3%)	0.928
	No	213 (71.0%)	212 (70.7%)	
Shortness of breath	Yes	40 (13.3%)	102 (34.0%)	<0.001**
	No	260 (86.7%)	198 (66.0%)	
Diarrhea	Yes	40 (13.3%)	44 (14.7%)	0.638
	No	260 (86.7%)	256 (85.3%)	
Chest pain	Yes	115 (38.3%)	147 (49.0%)	0.008**
	No	185 (61.7%)	153 (51.0%)	

The prevalence of diabetic and non-diabetic participants who were satisfied with the CanSino vaccine showed that most of the non-diabetic participants, 164 (54.7%), were pleased, and 155 (51.7%) diabetics and 65 (21.7%) non-diabetic participants were extremely pleased with their vaccinations. Moreover, 145 (48.3%) diabetics and 71 (23.7%) non-diabetic participants had no opinion. No participants in either group expressed dissatisfaction, and a statistically significant difference was observed among them (p<0.001), as presented in Table 3.

**Table 3:** Association of diabetes mellitus and participants' satisfaction levels with the CanSino vaccine *Data have been represented as n(%); **p-value significant as <0.05.

Variable*		n(%)		p-value
		Diabetes mellitus		
		Yes	No	
Overall participants' level of satisfaction with the vaccine	Highly satisfied	155(51.7%)	65(21.7%)	<0.001**
	Satisfied	0(0.0%)	164(54.7%)	
	No opinion	145(48.3%)	71(23.7%)	
	Dissatisfied	0(0.0%)	0(0.0%)	

## Discussion

This study demonstrated the reported local and general adverse effects of the CanSino vaccine in patients with diabetes and non-diabetic participants. According to a study by Lee et al. [12], DM increased the probability of grade three to four adverse reactions, although most studies showed that people with DM were less likely to develop substantial side effects than healthy individuals after receiving the COVID-19 immunization. The most common local adverse effects are pain, swelling, and inflammation at the injection site, whereas the most typical general side effects are headaches, high body temperature, and tiredness. Most adverse reactions are minor, disappear rapidly after immunization, and do not interfere with normal activities. There were no fatalities in any of the patients, not even those with new-onset DM or hyperglycemic issues, who experienced quick symptom resolution with acceptable management. Our study was consistent with the above-mentioned research and revealed that diabetes increased the probability of systemic side effects such as fever, although it was also reported at a high frequency in non-diabetic participants. In addition, local side effects such as pain, burning, and swelling at the injection site were more frequently reported in diabetic patients. Fortunately, there were no serious side effects or hospitalizations.

Another study found that 77 children had received their second dose of the COVID-19 vaccine. One of the 16 (20.78%) people who received the CanSino: Ad5-nCoV vaccination reported injection site pain as a local adverse effect, but the remaining recipients reported confusion (12.50%), headache (6.25%), fatigue (12.50%), and lack of appetite (12.50%) as systemic side effects. [13]. Our study was not in agreement with the above-cited studies and indicated that approximately 600 diabetic and non-diabetic participants were vaccinated with the CanSino vaccine, and fever was the most frequently reported systemic adverse effect by 260 (86.75%) diabetics and 279 (93%) non-diabetic participants. In addition, local side effects, including burning 255 (85.0%), pain at injection site 200 (66.7%), and swelling at injection site 150 (50%), were also observed in diabetic patients.

A questionnaire-based survey was conducted among 502 participants who visited immunization clinics in various cities throughout Pakistan. Participants’ ages ranged from 50.8±20.3 years on average. Fifty-three percent of the individuals received both doses of the vaccination. The most frequent symptom was injection site pain (49.8%), followed by asthenia (43%), muscle pain (29.5%), and swelling (24.5%). Compared with men, women reported more symptoms. Following the initial dosage of the vaccine, individuals experienced more acute symptoms, which, for the majority of them, resolved within a week. The respondents’ attitudes toward the vaccine were mostly favorable. Of the respondents, 48.6% claimed they chose to get vaccinated on their own, 47.4% were confident in the vaccine’s effectiveness, and 79.9% advised others to do the same [14]. Our study was inconsistent with the above-reported studies and revealed that out of 600 participants, most were males with both diabetes and non-diabetics, with a mean age of 36.94 ± 11.97 and 40.50 ± 16.91 years, respectively. As far as the reported side effects are concerned, fever was the most commonly observed in both diabetic and non-diabetic participants, followed by burning and pain at the injection site, with a significant difference among both groups (p<0.001). Regarding vaccine acceptability, most participants were highly satisfied with the CanSino vaccine.

A different study demonstrated that getting older was strongly associated with higher post-vaccination symptoms (p<0.001). The risk of experiencing symptoms at the injection site and across the body increased with age (p<0.001) and was also associated with an increase in age (p<0.001). The presence of symptoms following vaccination was not associated with having previously had a COVID-19 infection (p>0.05). In addition, the prevalence of rare post-vaccination symptoms was more closely associated with the existence of a comorbid disorder (p<0.001) than with injection site and systemic side effects. In comparison with the second dose of the vaccine, only the first dose produced noticeably higher pain, swelling, or redness at the injection site (p<0.05) [15]. Likewise, another study evaluated the knowledge and acceptance of the COVID-19 vaccine among Pakistani citizens. It is crucial to comprehend the vaccine’s significance in Pakistan because of the nation’s size, high prevalence of illnesses, relatively significant vaccine reluctance, and poor vaccination coverage [16]. Consequently, it is crucial to understand the general population’s behavior and acceptance rate to eradicate any obstacles to vaccination. These findings were corroborated by the outcomes of the present study and indicated that most of the participants belonged to the middle-aged group, and increasing age probably significantly increased the chances of reporting local and general side effects. The existence of comorbidities such as hypertension instead of diabetes was insignificantly associated with the reported side effects (p=0.806). Local adverse effects of vaccines such as pain, redness, and burning were more frequently reported, with a substantial difference between diabetic and non-diabetic participants (p<0.001).

Likewise, the survey findings show that receiving the vaccine by injection also resulted in the development of specific indications and symptoms. Over 50% of individuals reported symptoms such as rashes, redness, discomfort, and swelling at the injection site [14]. A survey conducted in Zimbabwe, where respondents expressed comparable concerns, produced comparable results [17]. The most often reported symptoms of additional whole-body problems were primarily after receiving a second dose. These symptoms include general fatigue, aches and pains in the muscles, lightheadedness, vomiting, and a temperature. In contrast to these findings, a study found that fever, followed by dyspnea and flu-like symptoms, was the most often reported symptom [18]. Because of the underlying bad health conditions that can exacerbate the symptoms, it is acceptable to assume that the frequency of symptoms increases with age. These findings were partially similar to those of the present study and showed that fever was the most commonly experienced general side effect after receiving the CanSino vaccination among diabetics as well as non-diabetics, followed by anxiety. Regarding local side effects, pain and burning at the injection site, followed by pain, were significantly more frequently reported in diabetic patients. These side effects were more prevalent in males than in females.

This study had a few limitations, including a cross-sectional design and self-reported side effects. The self-reporting of the data gives credence to its objectivity. Moreover, as the duration of monitoring was only six weeks, the long-term side effects of the vaccine couldn't be determined in our study. Additionally, in our study, males outnumbered females, and as many of the side effects of vaccines are sex-dependent, it was another limitation of our study. It is necessary to take specific actions to increase the immunization program in Pakistan to alleviate the morbidity and mortality rates associated with COVID-19.

## Conclusions

This study concluded that participants with comorbid diseases such as DM had both general and local side effects far more frequently than those without DM; however, the overall side effects were not lethal, although a minority of cases experienced serious side effects, such as dyspnea. The most noticeable side effects after a single dose of CanSino were fever, injection site pain, and burning. The CanSino vaccine did not require hospitalization and had a relatively low frequency of local and systemic side effects. Participants generally expressed high satisfaction and good tolerability with the CanSino vaccine. More cohort studies are advisable to explore the long-term negative effects of COVID-19 vaccinations.
